# Living with a robot at home: the complexity of living with assistive robots in everyday life

**DOI:** 10.3389/frdem.2026.1843555

**Published:** 2026-07-10

**Authors:** Lillian Hung, Donna Case, Nathan Velazquez, Karen Lok Yi Wong, Olga Petrovskaya, Lily Haopu Ren, Katrina Leigh Jang

**Affiliations:** 1 Innovation in Dementia and Aging (IDEA) Lab, School of Nursing, University of British Columbia, Vancouver, BC, Canada; 2 College of Health Sciences, The University of Michigan-Flint, Flint, MI, United States; 3 School of Nursing, University of Victoria, Victoria, BC, Canada; 4 Gerontology Research Center, Simon Fraser University, Vancouver, BC, Canada

**Keywords:** Actor-Network Theory, aging, dementia, domestication theory, quality of life, assistive robots, social connection, technology

## Abstract

**Introduction:**

The growing use of assistive robots in aging care raises important questions about how these technologies are experienced and integrated into everyday life. This study explores the use of two assistive robots, a telepresence robot (Double) in long-term care homes in Canada and a delivery robot (Labrador) in community-based settings in the United States, drawing on insights from two qualitative studies.

**Methods:**

Guided by Actor-Network Theory and domestication theory as sensitizing frameworks, we examine how robots are understood, used, and negotiated within the daily lives of older adults, family caregivers, service providers, and care staff. Data were drawn from semi-structured interviews, ethnographic observations, and team-based critical reflection sessions involving researchers from both projects. Reflexive thematic analysis was conducted to interpret experiences across both studies.

**Results:**

Three themes were identified: (1) robots as relational agents shaped by socio-material networks, (2) configuring human-robot relationships in everyday contexts, and (3) ethical and practical tensions related to implementation and withdrawal. Findings suggest that the integration of robots into care is not a linear process but involves ongoing negotiation among users, technologies, and environments. Rather than replacing human care, robots can mediate and transform relationships, enabling new forms of connection and support. At the same time, their use raises critical ethical considerations, particularly regarding sustainability, dependency, and equity.

**Discussion:**

By applying theoretical lenses to empirical cases, this study contributes to a more nuanced understanding of assistive robots as embedded in complex social and material practices, with implications for future research, design, and implementation in aging care.

## Introduction

The global population is rapidly aging. According to the World Health Organization (World Health Organization, 2025), in 2020, one in eight people worldwide was aged 60 years or above, and by 2030, this will increase to one in six. Canada and the United States are experiencing similarly accelerated demographic shifts. In Canada, 18.9% of the population was aged 65 or above in 2023, and this proportion is projected to rise to 21.4–23.4% by 2030 (Statistics Canada, 2024). In the United States, 17% of the population was aged 65 or above in 2022, with projections indicating an increase to 21% by 2030 (United States Census Bureau, 2020). This demographic transformation is reshaping health and social care systems, intensifying demands for innovative approaches to support older adults’ independence, well-being, and social connection.

In response, there has been growing interest in the use of assistive technologies, including robots, in aging care across home, community, and long-term care (LTC) settings. Sawik et al. (2023) conducted a review of 21 different types of robots for the care of older adults, identifying their diverse applications in daily tasks, physical assistance, social connection, and companionship. Mobile, humanoid, and pet robots are the most common types of assistive robots in supporting older adults, including daily tasks (e.g., cleaning, medication reminders), physical assistance, social connection, and companionship (Sawik et al., 2023). Further, Niemelä et al. (2021) classified seven types of robotic application services for different contexts and purposes, including maintaining independence and participation of older adults and supporting care work (Tuisku et al., 2023). A distinction is often drawn between social robots, which prioritize companionship and psychosocial support, and service robots, which focus on task completion and physical assistance. Understanding this distinction is important, as the functions and embodiments of robots shape how they are perceived and integrated into care practices.

A growing body of research has examined the usability, feasibility, and acceptability of assistive robots among older adults, family caregivers, and healthcare providers. Studies have demonstrated both the potential benefits of these technologies, such as supporting psychosocial well-being, enhancing independence, and facilitating care and the challenges associated with their use, including technical limitations, usability barriers, and ethical concerns related to privacy, surveillance, and the potential reduction of human contact. For example, Olde Keizer et al. (2019) investigated the use of NAO, an assistive robot designed to support health monitoring and physical training, among community-dwelling older adults in Netherlands. Their study focused on the usability of robots from an older adult perspective. To examine this, they observed and recorded interactions between the participants and the robot, and asked participants to complete a System Usability Scale, a subjective 10-item Likert scale. They identified several usability challenges, such as the robot’s difficulty in recognizing local dialects in the older adults’ speech, which hindered smooth interaction. Gasteiger et al. (2022) examined Bomy, a daily care robot that provides reminders for daily activities and offers cognitive games, among older adults living in retirement villages in New Zealand. Using interviews, they explored participants’ perceptions of the robot’s usefulness and feasibility. They found that older adults generally enjoyed interacting with robots as companions; however, technical problems, such as unresponsive screens during gameplay, affected their overall use. Hofstede et al. (2025) studied two social assistive robots, Lizz and Misty, with older adults living at home in Italy, Switzerland, and Netherlands. The robots provided reminders about daily activities, such as medication taking, which were set by caregivers. The researchers explored the acceptability of these robots among older adults, as well as formal and informal caregivers, through interviews. They found general acceptance but noted that participants, both older adults and caregivers, expected more interactive features. Ethical concerns were minimal among older adults but more pronounced among caregivers. Informal caregivers worried about data privacy and security, while formal caregivers expressed concern that robots might eventually reduce human contact. Beyond home and community settings, similar research has been conducted in long-term care facilities. Papadopoulos et al. (2022) carried out a randomized controlled trial in England and Japan to evaluate how Pepper, a humanoid assistive robot, could support the psychosocial well-being of residents. Participants, who were from English, Indian, and Japanese backgrounds, interacted with the robot through their activities of choice, which included conversation, music, video games, and family communication. The findings indicated that the robot enhanced emotional and mental well-being, particularly when its features were culturally responsive (e.g., playing culturally familiar music).

Recent research has explored the implementation of automated systems in support of older persons across various care settings. For example, Potter et al. (2026) have examined the deployment of assistive technologies in home environments, highlighting the importance of contextual factors and user engagement in successful adoption. These studies underscore the need for approaches that attend to the situated and relational nature of technology use in care settings. While this literature provides valuable insights into user experiences, it often remains descriptive and under-theorized.

The limited engagement with theory in this area represents a significant gap. Without theoretical grounding, it is difficult to fully understand the complex, relational, and contextual nature of human–technology interactions in care settings. The concept of praxis is widely discussed across social work (Samson et al., 2025) and occupational therapy (Restall, 2024). Occupational therapy emphasizes the inseparability of theory and practice in generating meaningful and socially responsive knowledge. Engaging with theory allows researchers to move beyond surface-level descriptions and examine the relational, contextual, and socio-material dynamics that shape experiences with assistive technologies. Praxis emphasizes the integration of theory and action to generate meaningful and socially responsive knowledge and to enhance quality of life. Building on this understanding of praxis, this study draws on theoretical perspectives to interpret the lived experiences of people using assistive robots. These perspectives support a more nuanced understanding that attends to how experiences are shaped through interactions among users, technologies, and care environments. While this approach highlights relational and everyday practices, it also points to the influence of broader organizational and structural conditions, such as institutional constraints, that shape how assistive technologies are implemented and used.

To address this gap, this paper draws on Actor-Network Theory (ANT) and domestication theory as sensitizing frameworks to interpret the experiences of older adults, family caregivers, service providers and care staff interacting with assistive robots. ANT directs attention to the relational networks through which both human and non-human actors co-constitute action. Domestication theory focuses on how technologies are incorporated into everyday life and acquire meaning through use. Together, these perspectives combine to offer a complementary lens to examine how assistive robots are not merely tools, but participants in dynamic socio-material practices.

This paper presents a critical reflection based on two empirical projects: (1) a telepresence robot study conducted in LTC homes in Canada and (2) an assistive delivery robot study conducted in community-based settings in the United States. These projects were selected because they capture diverse yet complementary contexts of robot use, involving different types of technologies, care settings, and user groups. Both studies highlight the complex and evolving ways in which robots are integrated into everyday care practices, as well as the ethical and practical tensions that arise during their implementation and withdrawal. In this paper, insights from the two projects are brought together through an integrative, secondary thematic synthesis. Rather than treating the studies as separate cases, we draw out shared patterns across both contexts. By examining these cases through a theoretically informed lens, this paper aims to explore the complex experiences of living with assistive robots in everyday life and to generate insights that can inform future research, design, and implementation of robotic technologies in aging care.

### Theoretical foundation

The study draws on Actor-Network Theory (ANT) and domestication theory. ANT directs attention to the relational networks through which both human and non-human actors co-constitute action, while domestication theory focuses on how technologies are incorporated into everyday life and acquire meaning through use. Together, these perspectives offer a complementary lens to examine how assistive robots are not merely tools, but participants in dynamic socio-material practices.

ANT conceptualizes social life as emerging through networks of relationships among both human and non-human actors (Latour, 1996; Law, 2008; Sayes, 2014). From this perspective, technologies are not passive tools but active participants and are shaped by the networks in which they are embedded. Importantly, ANT does not treat agency as an inherent property of either humans or technologies; instead, agency is understood as an effect of relationships within heterogeneous networks. This means that rather than asking whether a robot “has” agency, ANT directs attention to how agency emerges through interactions among users, technologies, infrastructures, and institutional conditions. This perspective enables us to examine how assistive robots participate in care practices, and how their capacities depend on interactions with users, caregivers, infrastructures, and organizational contexts. A key insight from ANT is the concept of “translation” (Latour, 1996), through which actors’ interests are aligned to form stable networks. In the context of assistive robots, translation occurs when the interests of residents, family caregivers, staff, and organizational leaders align around the robot’s use, enabling its integration into care practices. When translation fails or networks become unstable, robot use may be disrupted or discontinued.

Complementing ANT, domestication theory focuses on how technologies are integrated into everyday life and acquire meaning through use. Rather than viewing technology adoption as linear, domestication theory conceptualizes it as an ongoing, dynamic process through which technologies are appropriated, adapted, and embedded within routines, relationships, and social contexts. This process has been described through phases such as commodification, objectification, incorporation, and conversion, which together capture how technologies move from novel objects to meaningful elements of daily life (Berker et al., 2006; Brause and Blank, 2020). Domestication theory highlights that technologies do not always become successfully integrated; they may also be resisted, repurposed, or abandoned. The concept of “anticipatory domestication” has been used to describe how users imagine and prepare for technology use before it occurs, a process relevant to our United States study, where participants engaged with a prototype robot through a demonstration.

ANT and domestication theory address different but complementary analytical questions. ANT helps explain how robots come to influence care practices through networks of relationships among people, technologies, infrastructures, and institutions. Domestication theory, in contrast, focuses on how technologies are interpreted, adapted, and integrated into everyday life. ANT directs attention to distributed agency and socio-material relations, whereas domestication theory illuminates processes of meaning-making and incorporation into daily routines. Together, these frameworks allow us to examine both how robots become consequential within care networks and how they become familiar, meaningful, or resisted within everyday practice.

ANT and domestication theory have been applied separately in previous robotics research. However, few studies have brought these perspectives together to examine both the relational networks through which robots become consequential and the everyday practices through which they become meaningful. The combined approach extends prior robotics scholarship by examining both network formation and everyday appropriation across distinct care contexts. A key contribution of this study is bringing together ANT and domestication theory, which are often used separately in robotics research. This combination helps us understand not only how robots become part of care practices through relationships with people, technologies, and care settings, but also how users interpret, adapt, and integrate robots into their everyday lives. By examining both anticipated and lived experiences across distinct care contexts, we provide a more complete understanding of how assistive robots become meaningful in aging care.

### Objective

This paper aims to explore how assistive robots are understood, used, and negotiated within the daily lives of older adults, family caregivers, service providers, and care staff across different care contexts. By drawing on ANT and domestication theory as sensitizing frameworks, we examine the relational and socio-material dynamics through which robots become integrated or fail to become integrated into everyday care practices. Specifically, we investigate: (1) how robots participate in care networks and shape care practices; (2) how users interpret, adapt, and integrate robots into their daily routines and relationships; and (3) the ethical and practical tensions that arise during implementation and withdrawal.

## Methods

### Study design

This study is a critical reflection drawing on data from two empirical research projects examining the use of assistive robots in aging care. We analyzed existing qualitative data alongside reflection sessions to explore how assistive robots are experienced and integrated into everyday life across different care contexts. Actor-Network Theory (ANT) and domestication theory informed the interpretation of the data as sensitizing frameworks.

#### The Canada project (telepresence robot in long-term care)

**Figure 1:**
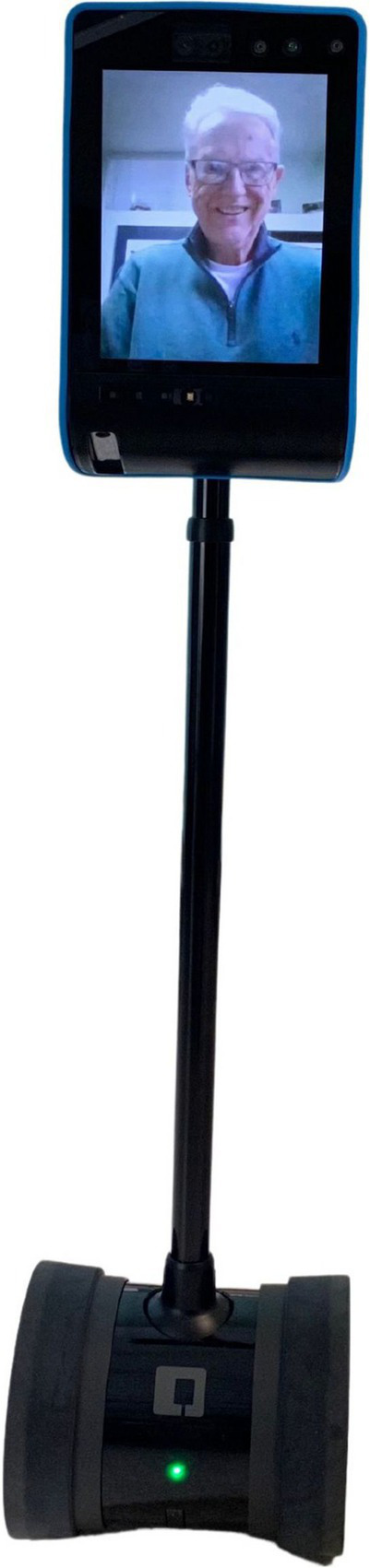
A double telepresence robot.

This study examined the implementation of telepresence robots (Double) across five long-term care (LTC) homes in British Columbia, Canada, between 2021 and 2024. The telepresence robot is depicted in Figure 1. A total of 20 robots were deployed to support virtual communication between residents and their family members. These robots were often stationed in residents’ rooms or designated areas of LTC homes. Family members could remotely initiate video calls, control the robot’s movement, and interact with residents without requiring staff assistance. No effort was needed by residents to be involved in video calls. The study focused on residents living with dementia, family caregivers, care leaders, and multidisciplinary frontline staff, including nurses, care aides, social workers, and occupational therapists. Participants were recruited using purposive and convenience snowball sampling. Inclusion criteria for residents included a diagnosis of dementia and the ability to communicate in English or Chinese. Family caregivers were eligible if they were connected to participating residents, and staff participants were required to be employed in frontline roles within the participating LTC homes. A total of 18 residents, 17 family caregivers, 5 care leaders, and 15 staff were included in the study. The research team deployed 20 telepresence robots across five LTC homes in British Columbia, Canada.

The study had four aims: (1) to explore staff perspectives on adopting telepresence robots in LTC homes; (2) to identify facilitators and barriers to implementation; (3) to examine the experiences of residents and family members using these robots; and (4) to evaluate the impact of telepresence robots on residents and family caregivers. Data collection and analysis were guided by the Consolidated Framework for Implementation Research (CFIR) and the Caregiver-centered Care Model.

#### The United States project (assistive delivery robot)

**Figure 2:**
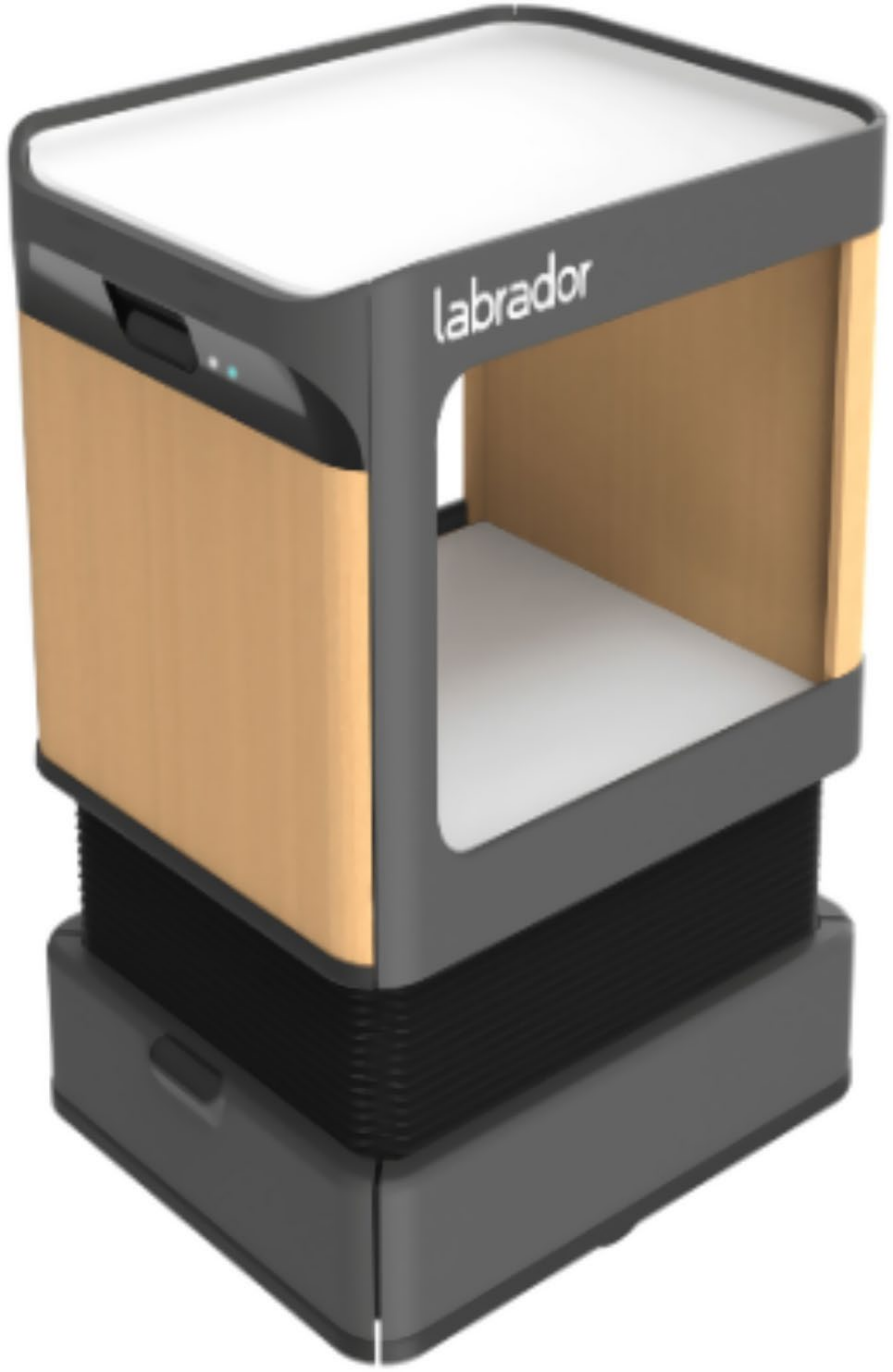
The labrador retriever system assistive robot.

The Labrador Retriever System™ project was conducted between 2021and 2024. See Figure 2. The Labrador Retriever System™ was presented to participants in a researcher-facilitated demonstration context rather than as an extended in-home deployment. The purpose of the demonstration was to support participants’ understanding of the system and to elicit perceptions and anticipated use cases following observation of the robot’s functions. The study did not involve iterative co-design sessions with participants during the data-collection encounter, nor did it assess real-world adoption, effectiveness, or outcomes during routine use. The study explored perceptions and potential uses of the Labrador Retriever System™, a programmable assistive robot designed to deliver items (e.g., medication, food, personal belongings) within home environments. The study took place in a Program of All-Inclusive Care for the Elderly (PACE) and a private non-profit independent living facility in southeast Michigan. Participants included older adults (aged 55 and older), caregivers (formal and informal), and service providers. Thirty-three participants were recruited through convenience sampling during robot demonstration sessions. Surveys were administered verbally by trained research team members, who also entered responses into Qualtrics. As the research team introduced the project and conducted the demonstration and survey, their presence may have shaped participants’ responses (e.g., social desirability or acquiescence effects). Accordingly, findings are interpreted as situated perceptions reported immediately after a demonstration, rather than as evidence derived from naturalistic, unsupervised use of the robot. This study provides data on anticipated rather than lived domestication. These data are valuable for understanding how potential users imagine integrating robotic technologies into their care arrangements, but they do not provide evidence of sustained use or real-world outcomes.

The two studies provide complementary rather than equivalent forms of evidence. The Canadian study examined the lived integration of telepresence robots in long-term care settings over time, exploring how robotic technologies became embedded in everyday practices and relationships. The United States study explored participants’ perceptions and anticipated use of a delivery robot following a structured demonstration. Bringing these studies together enabled us to examine both lived domestication and anticipated domestication. While one study reveals how human–robot relationships were enacted in practice, the other illuminates how potential users imagine integrating robotic technologies into future care arrangements. Examining both perspectives provides a broader understanding of how expectations, meanings, and care practices surrounding assistive robots are formed and negotiated.

We aimed to (1) explore older adults’ and caregivers’ perceptions of the usefulness of the system and to identify factors that allow older adults to remain in their homes as they get older and (2) examine older adults’ and caregivers’ perceptions of the use of the robot, and identify contextual factors that decrease older adults’ dependency on formal and informal caregivers.

### Data sources

#### Canada study data

The Canada study collected data through semi-structured interviews and focus groups with LTC residents with dementia (*n* = 18), family caregivers (*n* = 17), care leaders (*n* = 5), and multidisciplinary frontline staff (*n* = 15, including nurses, care aides, social workers, and occupational therapists). Ethnographic observations of robot use in care settings were conducted by the Principal Investigator (PI), Co-PI, and multidisciplinary trainees over 3 years (2021–2024). Fieldnotes documented implementation processes, training sessions, and everyday robot use. Research team meeting notes and debriefing sessions were included as part of the data source as well.

#### United States study data

The United States project collected data through researcher-facilitated robot demonstrations to 33 participants (older adults aged 55+, formal and informal caregivers, and service providers). One-time verbally administered surveys (5–10 min) contained both closed- and open-ended questions. Open-ended survey responses captured participants’ anticipated uses of the robot and perceived fit with their care needs. Demonstration feedback notes were recorded by the research team.

#### Team-based reflection data

Data also included transcripts from five team-based reflection sessions involving researchers from both projects. Each session lasted approximately 1 hour and was conducted virtually and audio-recorded. These sessions focused on critically examining experiences, patterns, and tensions observed across both studies. All authors participated in these sessions, which were facilitated by DC and LH. NV, KW, and LR contributed by linking empirical data to theoretical frameworks, while OP and KJ, who were not directly involved in either project, provided additional theoretical and clinical perspectives. All sessions were transcribed verbatim.

In total, the data corpus includes interview transcripts, focus group transcripts, survey responses, field notes, observational records, and reflection session transcripts. Detailed descriptions of study design, sampling, and data collection procedures for each study have been reported in prior publications (Hu et al., 2025; Wong et al., 2023; Ren et al., 2024; Case et al., 2023; Case et al., 2022).

### Data analysis

We analyzed the reflection data using Braun and Clarke’s (2006, 2019) reflexive thematic analysis. While ANT was not adopted as an initial theoretical framework in the design of the original empirical studies, it was applied in this analysis as a sensitizing lens to examine how agency, action, and outcomes emerged through relationships among human and non-human actors. Initial coding and theme development were conducted inductively, grounded in the empirical data across both projects. The research team moved iteratively between interview transcripts, observational field notes, reflection session transcripts, and emerging interpretations. ANT and domestication theory were then used as sensitizing frameworks to support interpretation of the emerging themes. Rather than imposing predefined categories, these theoretical perspectives informed how patterns in the data were interpreted, particularly in relation to relational agency, networked interactions, and the evolving integration of technology into everyday life.

Our analytic process involved the following steps: (a) five team members from the two projects (Telepresence Robot project: LH, KW, LR; Assistive Robot project: DC, NV) familiarized themselves with the data from both projects and the reflection session transcripts; (b) three trainees (NV, KW, and LR) coded the data and discussed and resolved any coding disagreements among themselves; (c) the three trainees generated initial themes; (d) the whole team reviewed the preliminary themes and discussed the relationships among them; (e) the team refined and reached consensus on the final themes; and (f) throughout the analytic process, the team discussed alternative interpretations and sought to ensure that themes remained closely grounded in participants’ accounts and observational data.

### Rigor

To ensure rigor, five team members from the two projects provided rich descriptions of each study and adopted two widely recognized theories, ANT and domestication theory, to guide our reflection. Team members from diverse professional and cultural backgrounds practiced reflexivity and challenged each other’s assumptions throughout the reflection process. LH, the PI of the Telepresence Robot project, is a senior researcher in nursing. DC, the PI of the Assistive Robot study, is a senior researcher in occupational therapy (OT). OP, a nursing researcher who uses ANT to study care practices involving medical digital technology, contributed to theoretical and practical discussions as an outsider to both projects. Three trainees, NV, KW, and LR are PhD students in occupational therapy, interdisciplinary studies, and social work, respectively. KJ is an occupational therapist with clinical experience in geriatric care. We also recognize that researchers played an active role in shaping the contexts within which participants encountered the robots. In the Canadian study, researchers supported implementation, training, and ongoing evaluation activities. In the United States study, participants were introduced to the robot through a researcher-facilitated demonstration and survey process. Consequently, participants’ perceptions may have been influenced by the technology’s framing, the demonstration context, and the presence of the research team. We therefore interpret participants’ responses as situated accounts that emerged through these research encounters rather than as neutral reflections of future adoption. This paper was written following the Consolidated criteria for Reporting Qualitative research (COREQ) checklist. See Supplementary material for the checklist.

## Results

We identified three themes: (1) robots as relational agents, (2) configuring human-robot relationship, and (3) ethical and contextual implications. Table 1 summarizes the themes and subthemes.

**Table 1:** Summary of themes and subthemes.

Themes	Subthemes
1. Robots as relational agents	1.1. Meeting diverse needs of older adults
	1.2. Supporting caregivers in care delivery
2. Configuring human-robot relationships in everyday contexts	2.1. Configuring human-robot relationships
	2.2. Innovative robot usage
3. Ethical and contextual tensions	3.1. Emotional attachment and withdrawal
	3.2. Environmental challenges and organizational conditions

### Theme 1: robots as relational agents

Across both studies, robots did not function as passive tools but played a role in care practices in ways that were shaped by the networks in which they were embedded. Their capacity to “act” depends on interactions with users, caregivers, infrastructure, and organizational conditions. Examples were shown in the subthemes below.

#### Meeting diverse needs of older adults

In the Canada study, the telepresence robot enabled residents to maintain social connections through remotely facilitated, real-time interactions. For example, after the research team introduced the robot to a female resident with dementia and her daughter, the daughter incorporated virtual visits into her daily routine. These visits depended on a stable internet connection, a charged and functioning robot, the daughter’s ability to remotely log into the system and navigate the robot and staff support in ensuring the robot remained available and appropriately positioned. Through the robot, the daughter communicated with her mother in Cantonese, guided her through simple exercises, and acknowledged her efforts. In another interaction, a resident remarked that the sunset outside her room was beautiful and wished to share the moment with her daughter. The daughter remotely navigated the robot toward the window and participated in the experience in real time. The resident later reflected, “I felt really good because she could join me and we enjoyed the sunset together.” Rather than being produced by the robot alone, this moment emerged through the coordinated work of family members, staff, technological infrastructure, software, and the physical environment.

While these robot-facilitated interactions were observed in the Canada study, similar patterns emerged in the United States study, where participants reflected how the robot could support everyday routines despite different contexts and functionalities. In the United States study, participants engaged with the assistive robot primarily through a brief demonstration followed by a one-time, verbally administered 5–10-min survey containing both closed- and open-ended questions (e.g., “How might you use this Labrador in your setting/daily activities?” and “When might you use it?”). Accordingly, the United States data do not document sustained use or downstream modifications to the system; rather, they capture participants *anticipated* uses and perceived fit immediately after observing the robot.

To avoid overstating what these data can support, we interpret these accounts as expressions of *anticipated appropriation*, how participants imagine integrating the robot into existing care routines, rather than as evidence that users “reshaped” the technology in practice. Participants’ suggestions were typically anchored in lived experiences (e.g., post-surgical recovery, fear of falling, medication routines) and remained broadly aligned with the system’s demonstrated purpose as a programmable item-delivery robot within the home (e.g., bringing medications, food, or personal items on a scheduled or requested basis). Where participants extended beyond the core demonstration (for example, emphasizing timed medication reminders via scheduled delivery), these suggestions are treated as perceived needs and desired functions, not as confirmed divergences from designers’ intentions or as changes that would necessarily be implemented.

At the same time, participants frequently noted that any such use would depend on additional human and material arrangements: items would still need to be prepared and placed onto the robot, and the system required programming, maintenance, and home spatial configuration. From this perspective, the robot was understood not as an autonomous solution, but as one element within a broader network of care that includes caregivers, household environments, and technological infrastructure. Unlike static objects such as newspapers, memory games, or stuffed toys, the telepresence robot enabled mobile, reciprocal, and real-time interactions across distance. Its ability to move, transmit audiovisual communication, and be remotely controlled allowed it to participate in social interactions in ways that other care objects could not. This distinction helps explain why participants often described the robot not simply as a device, but as an active part of their social and care experiences.

#### Supporting caregivers in care delivery

Robots also played a role in reshaping caregiving practices. In the Canada study, telepresence robots facilitated ongoing communication between residents and family caregivers, which in turn influenced care delivery. For example, staff reported that regular virtual visits by family caregivers helped some residents with dementia feel accompanied and cared for by their families. This sense of presence reduced feelings of loneliness and agitation among these residents. As a result, staff observed improved cooperation during care activities. In addition, robots enabled new forms of interaction and strengthened connections between family caregivers and staff. In one case, a family member initiated a video call while a staff member was providing care to the resident, with staff ensuring that the interaction respected the resident’s privacy. Through the robot, the family member was able to observe the care being delivered and expressed appreciation to the staff for their efforts. One staff member reflected: “When the family member was able to see what we were doing and thanked us afterwards, it felt really meaningful and motivating. It reminded me that families notice the care we provide, even when they cannot be physically present.” These examples show that the robot enabled new forms of communication and visibility within care practices, connecting residents, family members, and staff in ways that were not previously possible. In these situations, the robot participated in a broader network of mediation involving family members, staff, infrastructure, and organizational support.

Similarly, participants in the United States study described how the assistive robot could support the redistribution of caregiving activities, reflecting a comparable reconfiguration of care roles despite differences in care needs, contexts, and robot design. Routine tasks such as retrieving items, delivering water, or supporting medication schedules were seen as areas where the robot could reduce the frequency of caregiver involvement. For example, one participant reflected on relying heavily on a family member during post-surgical recovery and suggested that the robot could reduce the need to repeatedly call for assistance with small but frequent tasks. However, participants also emphasized the limits of the robot’s role. Care activities requiring physical assistance, such as bathing, dressing, or transferring, remained firmly within the domain of human caregivers. These reflections highlight how participants positioned the robot within existing care arrangements, envisioning it as augmenting rather than substituting care. In this sense, the robot contributed to a reconfiguration of caregiving practices; where certain forms of labor could be delegated to technology and others remained relational and required human intervention.

### Theme 2: configuring human-robot relationships in everyday contexts

#### Configuring human-robot relationships

In both studies, relationships between users and robots developed over time. These relationships could include active engagement, as well as forms of non-use or disregard. Therefore, rather than describing human-robot relationships as “evolving,” it is more accurate to describe them as “configuring,” or stabilizing into specific forms depending on the network configuration. Initial uncertainty or unfamiliarity often gave way to more active and personalized engagement as users became accustomed to technology.

In the Canada study, family caregivers were initially trained to operate the telepresence robot, including controlling its movement and initiating calls. Over time, these interactions became more fluid and integrated into daily routines. Family members used the robot not only for conversation but also to share music, artwork, and family events. For example, one daughter played the ukulele for her father during a virtual visit, while another facilitated a birthday celebration involving multiple family members joining simultaneously.

The robot was also incorporated into collective activities within LTC homes. Staff decorated the robot during holidays such as Halloween and Valentine’s Day, integrating it into social events and routines. These practices reflect how the robot acquired symbolic and social meaning within the care environment.

Similarly, in the United States study, human–robot relationships were configured through processes of interpretation and personalization, even when participants had limited direct experience using the robot. This suggests that relational engagement can begin through anticipation and imagination as well as through using the robot in practice. Some described the robot in relational terms, such as a “helper” or “pet,” and suggested naming it, indicating early stages of meaning-making. Others envisioned adapting the robot’s programmed routes (dubbed “bus stops”) to reflect their home layouts and routines.

**Figure 3:**
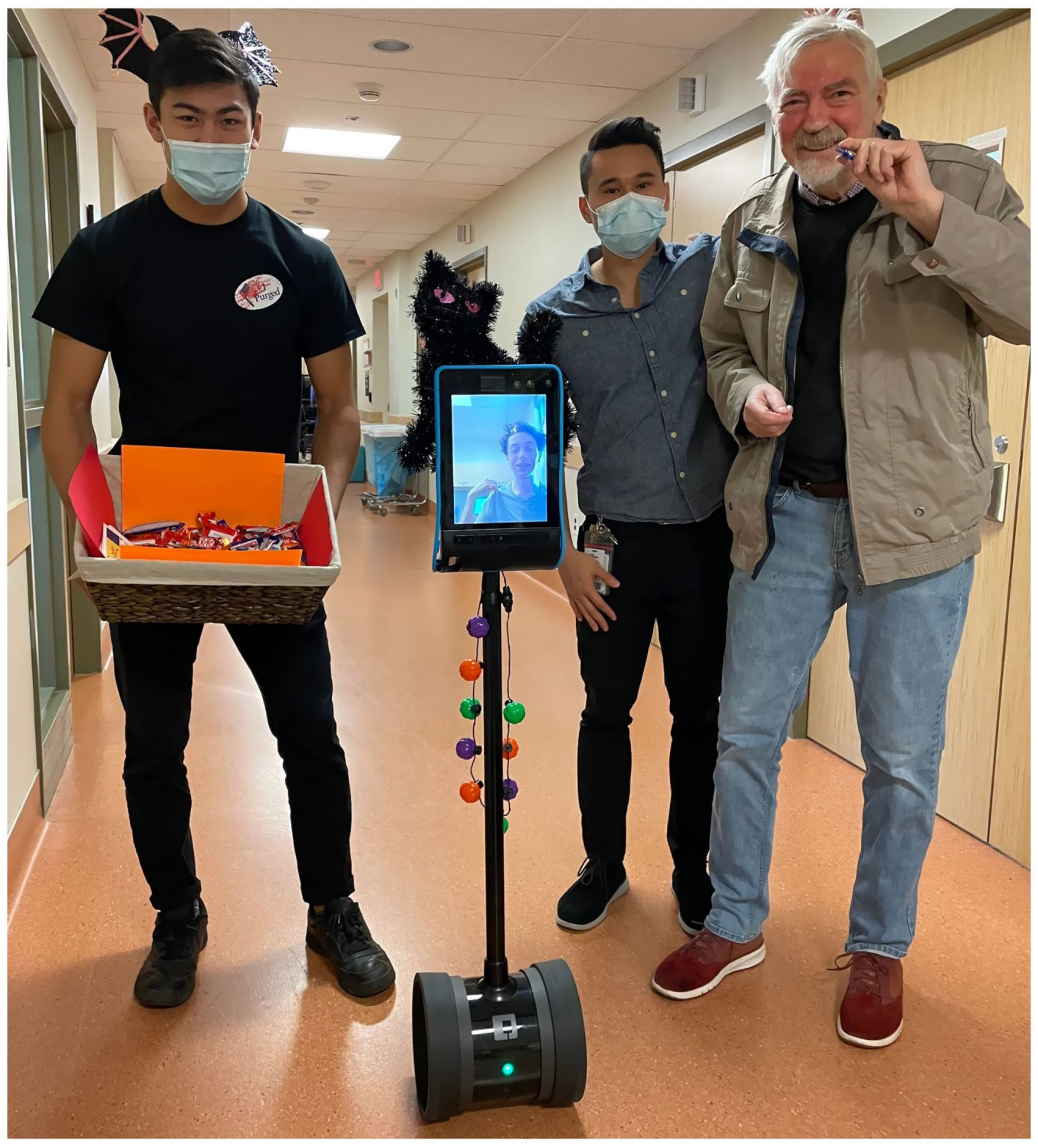
Telepresence robot incorporated into a Halloween activity in a long-term care home, illustrating its adaptation into social and communal practices. Such incorporation reflects domestication theory’s emphasis on how technologies acquire meaning through integration into cultural and relational practices.

**Figure 4:**
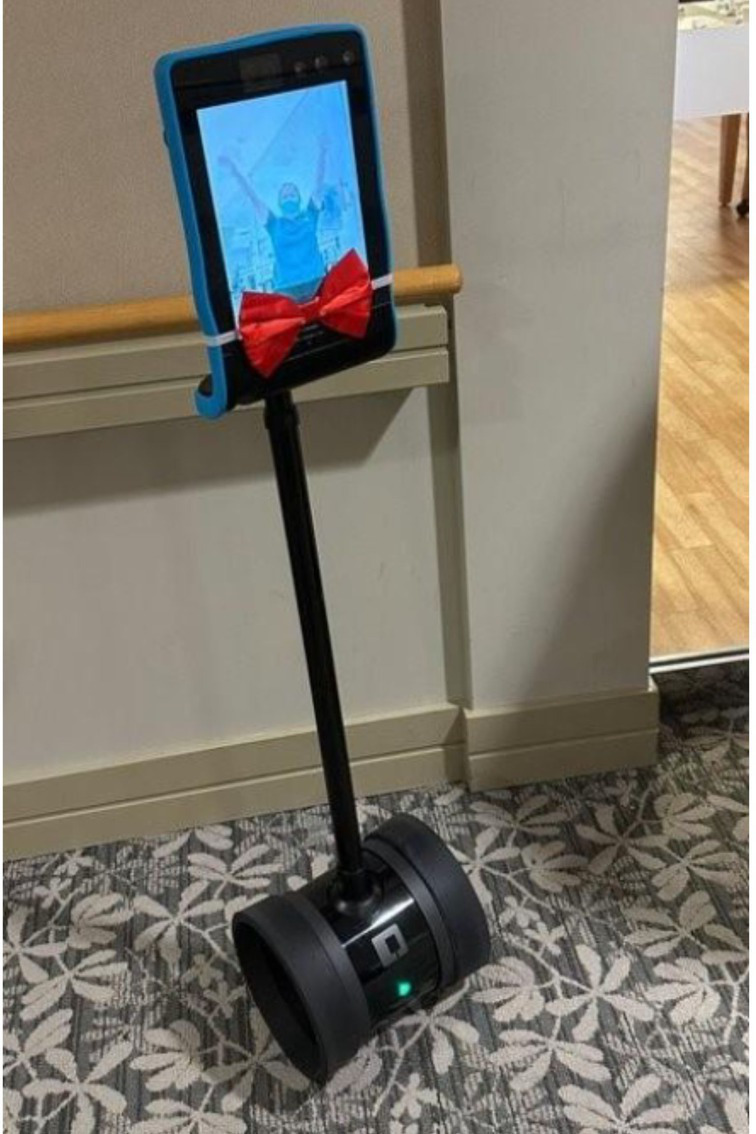
Telepresence robot used during a Valentine’s Day celebration, demonstrating its role in facilitating shared experiences between residents and family members. This illustrates how robots can become embedded in social rituals and acquire symbolic meaning beyond their functional purpose.

These reflections suggest that even in the absence of long-term use, participants began to domesticate the technology conceptually imagining how it could align with their identities, preferences, and daily practices. This highlights that domestication is not only a process of use but also anticipation and interpretation. The integration of the robot into everyday life extended beyond individual interactions and became embedded in collective and symbolic practices within LTC homes. Staff and trainees actively incorporated the robot into social events, reinterpreting its role beyond a communication device. For example, during Halloween (see Figure 3), staff dressed the robot as a child and used it to participate in “trick-or-treat” activities with residents. Similarly, on Valentine’s Day (see Figure 4), the robot was decorated with a bowtie and used to facilitate shared celebrations between residents and their family members. These practices illustrate how the robot acquired social and symbolic meaning within the care environment and became part of communal rituals rather than remaining as a purely functional device. From a domestication perspective, such adaptations reflect how technologies are not only incorporated into routines but are also reimagined and embedded within cultural and relational practices.

#### Innovative robot usage

Across both studies, participants engaged with the robots in ways that extended beyond their originally intended functions. In the Canadian study, these extensions emerged through actual use and adaptation in practice. In the United States study, participants imagined how the robot might be adapted to support needs and routines beyond those demonstrated.

In the Canada study, one family member joined a resident’s acupuncture session virtually through the robot. During the session, the family member provided emotional reassurance, translated between the resident and the therapist, and contributed knowledge about the resident’s condition. This interaction supported more personalized and coordinated care, while also illustrating how the robot enabled new forms of participation. In another example, a family used the robot to organize a virtual gathering across multiple geographic locations. The resident, who had limited communication with others in the care home due to language barriers, was able to interact with family members simultaneously. These examples demonstrate how users actively reconfigured the robot’s role in response to their relational and contextual needs.

In the United States study, participants demonstrated comparable forms of innovativeness by proposing possible uses that extended beyond the robot’s intended functions. Some suggested integrating the robot with communication platforms, such as voice assistants, to facilitate contact with family members, particularly for individuals with mobility limitations. Others imagined using the robot to support the well-being of residents, including enabling leisure activities by playing music, and enhancing independence in daily routines by tracking periodic nutrient intake. Although these uses were largely hypothetical, they reflect the same underlying process of reinterpretation as participants actively reimagined how the robot might fit within their own needs and contexts. This aligns with domestication theory’s emphasis on users as active interpreters of technology who imagine, negotiate, and anticipate potential uses that may diverge from designers’ original intentions. Because participants had not used the robot longitudinally, these findings reflect anticipated adaptation rather than observed modification in practice.

### Theme 3: ethical and contextual tensions

#### Emotional attachment and withdrawal

In both studies, relationships between users and robots developed over time. Initial uncertainty or unfamiliarity often gave way to more active and personalized engagement as users became accustomed to the technology.

In the Canada study, some residents expressed emotional attachment to the robot, describing it as a “friend” or part of their daily life. For example, one resident expressed sadness about the robot’s removal, stating, “I have a friend and he left. I liked seeing people through him. Now he is gone.” In contrast, other residents expressed indifference, indicating that attachment depended on their habits and acceptance of different types of technology enabling their social connection, as well as the extent to which the robot was integrated into their routines. For example, a resident who enjoyed virtual visits with family through the robot felt that his life would not be affected after its withdrawal. He explained, “I could still call my family members with the phone and talk over there. The phone is just over here, next to my bed, and it is so easy, I just need to pick up the speaker and dial. I am used to the phone.”

In the United States study, some participants expressed a desire for extended access to the robot, not only to explore its functions but also to incorporate it more fully into their daily lives. For example, one participant repeatedly requested a longer trial period, stating, “Let me have it longer,” suggesting an emerging sense of value and curiosity about the robot’s role in their routine. Other participants framed the robot in relational terms, such as a “helper” or companion-like presence and described how it could support independence in meaningful ways. These responses indicate that even limited interaction can initiate processes of meaning-making and attachment, particularly when the technology aligns with individuals’ perceived needs or aspirations for aging in place. At the same time, these responses also raise ethical questions about expectation management. When participants imagine future benefits from a prototype or time-limited intervention, researchers and developers must consider how to communicate uncertainty about availability, affordability, and long-term support. At the same time, the nature and depth of attachment differed across the two studies. In the Canada study, sustained and repeated interactions allowed the robot to become embedded in everyday routines and relationships, contributing to stronger emotional connections. In contrast, in the U. S. study, attachment was more prospective and anticipatory, shaped by participants’ imagination of future use rather than current prolonged engagement.

#### Environmental challenges and organizational conditions

Findings from both studies suggested that the ability of robots to function effectively and to be sustained over time depended on a range of environmental, organizational, and infrastructural conditions. Across both studies, robots did not operate independently but were embedded within complex socio-technical arrangements that enabled or constrained their use.

In the Canada study, while findings indicated robot-facilitated interactions that supported residents’ social and emotional well-being, these interactions depended on the alignment of multiple elements and a series of coordinated human and material actions. When any of these elements or actions were disrupted, interactions could be delayed, interrupted, or not occur at all. For example, family members were required to operate the robot using a personal device, initiate calls, and navigate it within the care environment.

In addition, organizational factors such as leadership support, staffing capacity, infrastructure (e.g., internet connectivity), organizational priorities, and policies played a critical role in shaping implementation. Although some LTC homes recognized the benefits of using these robots, sustaining their use required ongoing coordination and resources. At the level of the physical environment, the robot’s movement within the LTC environment was also shaped by physical and organizational constraints. Navigation depended on stable internet connectivity and unobstructed pathways; cluttered hallways, closed doors, or limited physical space could restrict mobility. In practice, robots required periodic repositioning by staff, and when not in use, they needed to be docked or plugged in to remain functional. At the level of everyday practice, staff were not required to actively facilitate each interaction; however, their involvement remained necessary in indirect ways. For example, staff needed to ensure that robots were charged, positioned appropriately, and available for use. These tasks, while often invisible, became part of the everyday work of care. At the organizational level, decisions about whether to continue using the robots beyond the study period were influenced not only by perceived value but also by competing institutional demands. Some care homes expressed concerns about the additional workload required to maintain the robots, particularly in the context of staffing shortages and high care demands. Others considered how adopting such technologies aligned with organizational goals, such as innovation, accreditation, or public image, which suggests that the robot’s “success” was not solely determined by its functionality, but by how well it aligned with institutional priorities and available resources. These environmental, organizational and infrastructural conditions also reflect broader structural constraints within care systems, including staffing shortages, resource limitations, and institutional priorities that shape whose needs can be supported and how technologies are implemented. From a policy perspective, privacy policies varied across care homes. While some care homes allowed family members to remotely navigate the robot and accompany residents in shared spaces (e.g., hallways), other care homes expressed concerns about the potential privacy risks for other residents who might be visible through the robot during virtual visits. These variations in policy and practice also point to differences in institutional governance and accountability, highlighting how structural arrangements shape the environments and conditions under which technologies can be used, and to what extent.

These dependencies and considerations highlight that the robot’s capacity to “act” was contingent on ongoing human support, organizational practices, and environmental conditions. These situations also generated ethical tensions concerning fairness, responsibility, and access. Participants questioned whether technologies that improved quality of life would remain available after the study ended, while staff considered the resources required to sustain them. Ethical concerns emerged not as abstract principles but through everyday decisions about who could access the robots, who was responsible for maintaining them, and whose needs were prioritized when resources were limited. From an ANT perspective, these tensions arose through the socio-technical networks that shaped robot use, highlighting how ethical questions were intertwined with organizational, material, and relational conditions.

In the United States study, similar considerations emerged, although framed more prospectively. Participants highlighted the importance of technical support, affordability, and adaptability for long-term use. As the assistive robot was a prototype system, its operation depended on ongoing support from developers, particularly for configuring movement pathways (i.e., “bus stops”) within the home. Participants questioned whether such support would be sustainable over time, especially as their needs evolved with aging. In addition, the integration of the robot into home environments required spatial and relational adjustments. The robot needed to navigate within existing layouts, which may include narrow pathways, furniture constraints, or multi-level homes. It also required users or caregivers to take on new roles, such as programming task schedules, loading items, and troubleshooting issues. These responsibilities highlight that adopting assistive robots involves not only using a device but reorganizing aspects of daily life and care practices. Such reorganization may be experienced as empowering when it supports independence, but it may also transfer new forms of responsibility to older adults or informal caregivers. This creates an ethical tension between promoting autonomy and increasing burden. Across both studies, these findings point to the importance of viewing assistive robots as part of broader socio-technical systems. From an ANT perspective, the robot’s capacity to act depended on the stability and alignment of multiple elements, including human labor, infrastructure, institutional support, and technological design. When these elements were well-coordinated, the robot contributed to care practices; when they were disrupted or absent, the robot’s functionality and its perceived value declined. Importantly, findings from the United States study should be interpreted as accounts of anticipated domestication rather than evidence of actual adaptation or long-term integration. Participants described how they imagined incorporating the robot into future care arrangements, allowing us to examine expectations and projected meanings rather than enacted practices.

## Discussion

This study explored how assistive robots are experienced and integrated into everyday life across long-term care and community settings. Drawing on two empirical projects and guided by Actor-Network Theory (ANT) and domestication theory as sensitizing frameworks, we found that robots are not simply tools but participants in dynamic socio-material networks. Their capacity to support care and connection emerged through ongoing interactions among users, caregivers, infrastructures, and organizational contexts. Across both studies, robot use was characterized by three interrelated processes: (1) relational agency shaped by networks of human and non-human actors, (2) configuring human–robot relationships as technologies became integrated into everyday practices, and (3) ethical and practical tensions related to sustainability, attachment, and withdrawal. These findings highlight that the value and meaning of assistive robots are not fixed but continuously negotiated in context.

A key contribution of this study is to move beyond dominant framings of assistive robots as either functional tools or threats to human care. In contrast to the commonly invoked dichotomy between “warm human touch” and “cold technology,” our findings align with socio-material perspectives that understand technologies as being both functional and affective (Pols and Moser, 2009; Petrovskaya, 2023). This finding is consistent with Lipp’s (2023) analysis of how care emerges through human-machine interfacing, where care is enacted relationally rather than residing solely in either human or technological actors. In our study, robots were not experienced as replacements for human care, but as mediators of relationships. Residents described robots as companions or “friends,” while staff incorporated them into social and cultural practices, such as celebrations and shared activities. This challenges simplistic assumptions that technology inherently dehumanizes care. Instead, our findings suggest that robots can participate in and even enhance relational practices, depending on how they are embedded in everyday contexts. In the United States study, participants also imagined that delegating more routine retrieval tasks to the robot could shift family interactions away from task-based caregiving and toward more reciprocal familial relationships.

An important contribution of this study is the distinction between lived and anticipated domestication. In the Canadian study, robot use was observed as an ongoing practice embedded within everyday relationships, routines, and organizational conditions. In contrast, participants in the United States study engaged in anticipatory forms of domestication, imagining how the robot might fit within future care arrangements. While these anticipated uses do not provide evidence of actual adoption, they offer insight into how expectations, hopes, concerns, and perceived needs shape technology uptake before implementation occurs. This observation resonates with STS scholarship, demonstrating that expectations and imagined futures play an important role in shaping technology adoption long before technologies become embedded in everyday practice. Anticipatory engagements with technology can reveal how users negotiate potential benefits, concerns, and identities associated with future care arrangements. Examining both forms of domestication contributes to a more comprehensive understanding of how assistive robots become meaningful across different stages of technology integration. Importantly, findings from the United States study should be interpreted as accounts of anticipated domestication rather than evidence of actual adaptation or long-term integration. Participants described how they imagined incorporating the robot into future care arrangements following a structured demonstration. These accounts provide insight into expectations, perceived needs, and projected meanings associated with the technology, but they do not constitute evidence of sustained use, enacted practices, or observed modifications of the robot in everyday life.

The process of anticipated appropriation involved more than participants imagining future uses of the robot. The technology itself actively shaped these expectations through the functions, possibilities, and assumptions embedded in its design. In the United States study, participants’ responses were influenced not only by their personal experiences and care needs but also by the possibilities presented through the robot’s design and demonstration. Following ANT and STS perspectives, the robot can be understood as participating in the configuration of expectations, identities, and care arrangements even prior to sustained use. This observation resonates with Lipp’s (2023) work on how care comes to matter through human-machine interfacing and with Maibaum et al.’s (2022) critique of healthcare robotics, which highlights the importance of examining how design assumptions and implementation practices shape understandings of care and technological value. During demonstrations, participants frequently framed the robot as a means of promoting independence, reducing caregiver burden, and supporting aging in place. These expectations were influenced by the robot’s programmed capabilities and the ways in which those capabilities were presented. At the same time, participants often extended beyond the designers’ intended script, envisioning roles related to companionship, communication, and emotional support that were not central to the robot’s original purpose. Consistent with STS scholarship on technological scripts and care robotics, these findings suggest that anticipated appropriation emerges through a dynamic interplay between design intentions and users’ reinterpretations of what technologies might become within everyday care practices.

Drawing on ANT, this study advances an important conceptual clarification: assistive robots are not inherently agential but become so through relationships. Agency, in this sense, is not located within the robot itself, but is an effect of the network in which it is embedded (Law, 2008; Petrovskaya, 2023; West and Petrovskaya, 2025). ANT shifts attention away from asking whether robots are agents and instead toward examining how agency emerges through relationships among humans, technologies, infrastructures, and institutions. Instead, researchers should examine the conditions and networks through which a robot’s agency emerges as an effect. Although our empirical cases did not begin as ANT-informed studies, applying selected ANT concepts was useful for thinking through the available data. A similar recognition that successful application of assistive robots requires a “whole system” perspective (i.e., not limited to technology) can be provided by other theoretical frameworks (e.g., a logic model); in this way, our analysis echoes findings of other studies (Ryan et al., 2024; Allan et al., 2023). Perhaps one of ANT insights relevant here is that regardless of the degree to which robot use (and meaning) was intended by designers, its actual use and agency is an empirical question that can be usefully addressed through the ANT lens.

Notably, this relational understanding of agency is relevant for older adults living with dementia, whose cognitive capacities may vary or decline over time. In our study, engagement with the robots did not rely solely on individual cognition but was supported through relationships with family members, staff, and technological infrastructures. In this sense, agency was not diminished but reconfigured, as participation in social interaction and care practices was enabled through these socio-technical arrangements.

In addition, an important implication of these findings concerns the question of what constitutes meaningful care in the context of assistive technologies. In this study, meaningful care was not defined solely by the presence or absence of human contact, but emerged through participants’ experiences of connection, recognition, and support in everyday life. For residents and family members, meaningful care was often expressed through moments of togetherness, such as communicating in one’s preferred language, participating in routine activities together, or maintaining a sense of presence despite physical distance. At the same time, these findings also reveal ambivalences. While robots could strengthen relationships by facilitating ongoing communication and emotional connection, they also had the potential to reshape patterns of care, including reducing the frequency or necessity of in-person visits in some cases. From a care ethics perspective, this suggests that technologies do not simply add to existing care practices but reconfigure the networks of relationships through which care is enacted. Meaningful care, therefore, is not a fixed standard but a negotiated and context-dependent achievement, shaped by the interplay of human intentions, technological mediation, and structural conditions.

These findings highlight that robots do not act independently but through networks of relationships. Their ability to facilitate communication, shared experiences, and caregiving support depended on the alignment of infrastructure, human labor, organizational support, and user engagement. When these elements were present, robots contributed meaningfully to care practices; when they were absent, their usefulness diminished. Another key contribution of this study is to show how users actively reinterpret and, in some contexts, extend the functions of assistive robots beyond their intended design. In the Canadian study, these reinterpretations were observed in practice, whereas in the United States study they were expressed as anticipated possibilities following the robot demonstration. In both studies, participants adapted robots to fit their relational, cultural, and practical needs, from facilitating therapy sessions and family gatherings to integrating them into social rituals. This aligns with domestication theory, which emphasizes that technologies acquire meaning through use rather than through design alone. Users do not passively adopt technology; they actively interpret, negotiate, and sometimes rewrite technologies in ways that reflect their contexts and priorities. In our United States study, these processes were expressed through participants’ imagined and anticipated uses rather than through sustained interaction with the robot. In ANT terms, this can be understood as an “anti-program” (Bergschold, 2016), where the intended script of the technology is modified in practice. Importantly, this process was not limited to actual use. In the United States study, even brief exposure to the robot prompted participants to imagine how it could fit into their lives, suggesting that domestication begins not only through use but also through anticipation and interpretation. This highlights the importance of considering both lived and imagined interactions in understanding technology adoption.

A further contribution of this study is to foreground the often-overlooked conditions required to sustain assistive robots in real-world settings. Our findings show that robot use depends on a range of infrastructural, organizational, and relational factors, including internet connectivity, staff capacity, technical support, and alignment with institutional priorities. These findings resonate with Latour’s (1996) concept of the “translation of interests”, whereby technologies are adopted not solely for their technical capabilities but because they align with broader organizational goals, such as innovation, reputation, or efficiency. When such alignment is absent or breaks down, the networks supporting the technology fail to stabilize, resulting in partial use, discontinuation, or non-use. In our study, some care homes embraced robots as part of their innovation agenda, while others struggled to sustain them due to competing demands and limited resources. By highlighting these dynamics, this study shifts the focus from evaluating robots as isolated technologies to understanding them as components of complex socio-technical systems. This perspective helps explain why promising technologies often fail to be sustained in practice not because they lack value, but because the networks required to support them are fragile or misaligned. This perspective further shifts attention away from individual-level explanations such as user acceptance and instead foregrounds the relational and infrastructural conditions that enable or constrain technology use. It suggests that non-use and failure are not deficits of users or technologies alone, but outcomes of unstable or misaligned socio-technical arrangements.

Our findings provide a concrete illustration of this relational understanding of agency. For example, the telepresence robot’s ability to produce meaningful outcomes depended on a network of relationships that included internet connectivity in the LTC facility, staff willing to charge and position the robot, family members willing to operate it, and technical support to maintain the system. Staff described how the robot enabled new forms of communication and visibility between families and care providers. However, mediation was not performed by the robot alone. Rather, these interactions emerged through the alignment of multiple human and non-human actors, including residents, family members, staff, organizational support, internet connectivity, and the robot itself. Together, these actors reshaped relationships and contributed to changes in caregiving practices.

Domestication theory draws on the above idea that in their everyday life, users adapt or domesticate technology (Bergschold, 2016). According to Bergschold (2016), a branch of domestication studies that employs naturalistic observation of technology use in everyday life, is aligned with ANT. This branch of domestication studies is less concerned with the topic of consumption and more interested in how actual uses of technology might deviate from a designer’s prescribed vision, what ANT theorists call anti-program, or the users’ active re-writing of a “script” inscribed in the physical form and function of an object by its designers (Bergschold, 2016).

Our findings also surface important ethical tensions that emerge when assistive robots are introduced into care settings. First, the development of emotional attachment raises questions about the ethics of withdrawal. For some participants, the removal of the robot represented not just the loss of a device, but the disruption of meaningful relationships and routines. At the same time, other participants experienced little distress when the robot was withdrawn, highlighting that the perceived value of the technology varied across users and contexts. Second, ethical tensions emerged not only through participants’ emotional responses to robots, or the presence or withdrawal of the robots, but also through ethical decisions about access, allocation, and sustainability, which point to justice and equity in the allocation of resources and opportunities. While some participants benefited from robots during the study, not all could continue to access them afterward, raising questions about equity and responsibility. Issues of equity and access became evident when technologies could not be sustained beyond the research context. These concerns echo broader debates about the uneven distribution of digital and assistive technologies and the potential risk of exacerbating existing inequities in care (Roux, 2012). These tensions further challenge researchers, practitioners, and decision-makers to ask questions of: Who should bear responsibility for sustaining access to beneficial technologies—the individual, the care institution, or the technology provider? Third, the integration of robots into care practices introduces new forms of visibility and potential surveillance. For example, telepresence robots enabled family members to observe care in real time, which could both support transparency and reshape power dynamics between families and staff. These ethical considerations underscore the need for ongoing reflexivity in the design, implementation, and evaluation of assistive technologies, particularly in relation to power, responsibility, and social justice.

### Implications for practice, research, and ethics

This study has several implications. For practice, the findings suggest that successful implementation of assistive robots requires attention to the networks in which they are embedded. This includes ensuring adequate infrastructure (e.g., connectivity), supporting staff and caregivers in their roles, and aligning technology use with organizational priorities and workflows. Importantly, the work required to sustain these technologies, such as charging, positioning, and troubleshooting, should be recognized and supported. Critical analyses of the additional workload created by care technologies are a well-developed area of scholarship. Feminist scholars (Parks, 2010) point out how technology designed to support informal female caregivers occasionally worsens their quality of life. A literature review (Hamblin, 2022) argued that care robots and telecare and “smart” technology in the United Kingdom are producing new kinds of work and require “machine babysitters.” Another review (Mohammadnejad et al., 2023) found that information and communication technologies skyrocketing in healthcare, producing varied effects on nurses’ workload, frequently leading to obstacles in care. Authors in a special volume of *Social Science and Medicine* devoted to “how health professionals make digital technology work” (Petrakaki et al., 2025) argued that medical digital technology is often imposed upon health professionals and creates new uncertainties and inter-professional tension in their work.

For research, our study demonstrates the value of using theoretical frameworks such as ANT and domestication theory to move beyond descriptive accounts of technology use. Future research should examine how assistive technologies are embedded in everyday practices over time, with particular attention to how networks are formed, maintained, and disrupted. There is also a need to explore emerging forms of assistive technologies, including AI-enabled systems, while critically examining their relational and ethical implications. For ethics, our findings highlight tensions related to equity, sustainability, and responsibility. Not all users had equal access to or benefit from the robots, particularly after study completion. Questions arise regarding who is responsible for maintaining access to beneficial technologies and how decisions are made about their continuation or withdrawal. Additionally, while robots can enhance connection, they may also introduce new forms of surveillance or shift power dynamics within care relationships. Addressing these issues requires ongoing reflexivity and collaboration among researchers, practitioners, users, and technology developers.

### Limitations

This study has several limitations. First, ANT was applied in a pragmatic and non-purist manner as a sensitizing framework rather than a fully operationalized methodology. As a result, our analysis does not comprehensively map all network relations or power dynamics involved in robot use. Future research could employ ANT more systematically to trace the full range of actors and translations involved in robot implementation. Second, the two projects differed in design, context, and level of participant engagement, with the United States study relying more on short-term interactions and anticipated use. While this provided complementary perspectives, it also limits direct comparability between the cases. Third, the United States study’s data capture anticipated rather than actual use, which means our findings from that context reflect participants’ projections rather than their experiences of sustained engagement with the robot. Fourth, as a secondary analysis and critical reflection, the findings are shaped by the available data and the research team’s interpretations. The data were not originally collected with ANT or domestication theory in mind, which may have limited our ability to fully explore theoretical concepts. Fifth, the research team’s presence in both studies may have influenced participants’ responses, particularly in the United States study, where participants encountered the robot through a researcher-facilitated demonstration. We have interpreted participants’ responses as situated accounts that emerged through these research encounters rather than as neutral reflections of future adoption.

## Conclusion

This study offers a nuanced understanding of how assistive robots become embedded in the everyday lives of older adults across care settings. Rather than functioning as neutral tools, robots participated in relational networks that shaped care practices, social connections, and meanings of independence. Drawing on ANT and domestication theory as sensitizing lenses, we show that robot use is not determined solely by technological capability, but emerges through ongoing negotiation among users, caregivers, environments, and institutional conditions. Importantly, the findings challenge dominant concerns that robots inherently dehumanize care. Instead, they reveal how robots can foster new forms of relationality, while also introducing ethical tensions—particularly around withdrawal, sustainability, and equity. At the same time, these findings highlight that the benefits of assistive robots are contingent on the stability of the socio-technical networks in which they are embedded. When these networks are disrupted by conditions such as limited resources, infrastructural breakdowns, or misalignment of organizational priorities, robot use may be constrained or fail to be sustained. They also point to the importance of considering what constitutes meaningful care in this context, as experiences of care were shaped not only by technological mediation but by how relationships were maintained, reconfigured, or, at times, diminished. Future research should move beyond evaluating isolated technological features and instead examine how robots are situated within complex socio-material networks. Attending these relational dynamics is essential for developing technologies that are not only functional, but meaningful, ethical, and responsive to the lived realities of older adults.

## Data Availability

The raw data supporting the conclusions of this article will be made available by the authors, without undue reservation.

## References

[ref1] Allan H. T. Chris C. Mehigan S. Trueman S. (2023). Opening up conversation: collaborative working across sociomaterial contexts in nursing in London. J. Adv. Nurs. 80, 226–236. doi: 10.1111/jan.15799 37469168

[ref2] Bergschold J. M. (2016). Domesticating homecare services; vehicle route problem solver displaced. Nord. J. Sci. Technol. Stud. 4, 41–53. doi: 10.5324/njsts.v4i2.2184

[ref3] Berker T. Hartmann M. Punie Y. Ward K. J. (2006). Domestication of Media and Technology. London: Open University Press.

[ref4] Braun V. Clarke V. (2006). Using thematic analysis in psychology. Qual. Res. Psychol. 3, 77–101. doi: 10.1191/1478088706qp063oa

[ref5] Braun V. Clarke V. (2019). Reflecting on reflexive thematic analysis. Qual. Res. Sport Exerc. Health 11, 589–597. doi: 10.1080/2159676X.2019.1628806

[ref6] Brause S. R. Blank G. (2020). Externalized domestication: smart speaker assistants, networks and domestication theory. Inf. Commun. Soc. 23, 751–763. doi: 10.1080/1369118X.2020.1713845

[ref7] Case D. Velazquez N. Conti G. (2023). Will Older Adults Accept a Robotic Assistance system? A Preliminary Report. Flint, MI: Department of Occupational Therapy, University of Michigan-Flint.

[ref8] Case D. Velazquez N. Savich T. (2022). Perceptions of Older Adults and Caregivers of an Automated Robot to Facilitate Aging in Place. Indianapolis, IN: Gerontological Society of America Annual Conference.

[ref9] Gasteiger N. Ahn H. S. Fok C. Lim J. Lee C. MacDonald B. A. . (2022). Older adults’ experiences and perceptions of living with Bomy, an assistive dailycare robot: a qualitative study. Assist. Technol. 34, 487–497. doi: 10.1080/10400435.2021.1877210, 33544067

[ref10] Hamblin K. A. (2022). Technology in care systems: displacing, reshaping, reinstating or degrading roles? N. Technol. Work. Employ. 37, 41–58. doi: 10.1111/ntwe.12229, 35911255 PMC9304303

[ref11] Hofstede B. M. Askari S. I. Lukkien D. Gosetto L. Alberts J. W. Tesfay E. . (2025). A field study to explore user experiences with socially assistive robots for older adults: emphasizing the need for more interactivity and personalisation. Front. Robot. AI 12:1537272. doi: 10.3389/frobt.2025.1537272, 40270913 PMC12015597

[ref12] Hu G. Wong J. Ren L. H. Kleiss S. Berndt A. Wong L. . (2025). Care partner experience with telepresence robots in long-term care during COVID-19 pandemic. Digit. Health 11:20552076251319820. doi: 10.1177/20552076251319820, 39917415 PMC11800240

[ref13] Latour B. (1996). Aramis, or The Love of Technology. Cambridge, MA: Harvard University Press.

[ref14] Law J. (2008). “Actor network theory and material semiotics,” in The New Blackwell Companion to Social Theory, ed. Turner B. S. (Hoboken, NJ: John Wiley & Sons, Ltd), 141–158.

[ref15] Lipp B. (2023). Caring for robots: how care comes to matter in human-machine interfacing. Soc. Stud. Sci. 53, 660–685. doi: 10.1177/03063127221081, 35387514

[ref16] Maibaum A. Bischof A. Hergesell J. Lipp B. (2022). A critique of robotics in health care. AI Soc. 37, 467–477. doi: 10.1007/s00146-021-01206-z

[ref17] Mohammadnejad F. Freeman S. Klassen-Ross T. Hemingway D. Banner D. (2023). Impacts of technology use on the workload of registered nurses: a scoping review. J. Rehab. Assist. Technol. Engineer. 10:20556683231180189. doi: 10.1177/20556683231180189, 37342268 PMC10278405

[ref18] Niemelä M. Heikkinen S. Koistinen P. Laakso K. Melkas H. Kyrki V. (2021) Robots and the future of welfare services—A Finnish roadmap. Aalto University publication series. CROSSOVER. Available online at: http://urn.fi/URN:ISBN:978-952-64-0323-6 (Accessed January 2, 2026).

[ref19] Olde Keizer R. A. C. M. van Velsen L. Moncharmont M. Riche B. Ammour N. Del Signore S. . (2019). Using socially assistive robots for monitoring and preventing frailty among older adults: a study on usability and user experience challenges. Health Technol. 9, 595–605. doi: 10.1007/s12553-019-00320-9

[ref20] Papadopoulos C. Castro N. Nigath A. Davidson R. Faulkes N. Menicatti R. . (2022). The CARESSES randomised controlled trial: exploring the health-related impact of culturally competent artificial intelligence embedded into socially assistive robots and tested in older adult care homes. Int. J. Soc. Robot. 14, 245–256. doi: 10.1007/s12369-021-00781-x, 33907589 PMC8062829

[ref21] Parks J. A. (2010). Parks lifting the burden of women’s care work: should robots replace the “human touch”? Hypatia 25, 100–120. doi: 10.1111/j.1527-2001.2009.01086.x

[ref22] Petrakaki D. Chamakiotis P. Russell E. Charlwood A. (2025). Resistance, tensions and consent to digital working in healthcare. Soc. Sci. Med. 368:117691. doi: 10.1016/j.socscimed.2025.117691 39893068

[ref23] Petrovskaya O. (2023). “Technology and nursing,” in Routledge Handbook of Philosophy and Nursing, ed. Lipscomb M. (London: Routledge), 481–493.

[ref24] Pols J. Moser I. (2009). Cold technologies versus warm care? On affective and social relations with and through care technologies. Alter. 3, 159–178. doi: 10.1016/j.alter.2009.01.003

[ref25] Potter S. Hawley M. Higgins A. Amirabdollahian F. Dragone M. Di Nuovo A. . (2026). Assistive robotics for healthy aging: a foundational phenomenological co-design exercise. J. Med. Internet Res. 28:e77179. doi: 10.2196/77179, 41605501 PMC12895153

[ref26] Ren L. H. Wong K. L. Y. Wong J. Kleiss S. Berndt A. Mann J. . (2024). Working with a robot in hospital and long-term care homes: staff experience. BMC Nurs. 23:317. doi: 10.1186/s12912-024-01983-0, 38720346 PMC11080152

[ref27] Restall G. (2024). Mobilizing critical occupational therapy praxis to promote structural justice, equity, and rights. Canadian J Occupat. Ther. 91, 305–324. doi: 10.1177/00084174241277950, 39387131 PMC11852522

[ref28] Roux J. (2012). “Agencies’ democracy: “contribution” as a paradigm to (re)thinking the common in a world of conflict,” in Agency Without Actors?: New Approaches to Collective Action, eds. Passoth J. H. Peuker B. M. Schillmeier M. (London: Routledge), 130–154.

[ref29] Ryan T. Ryan N. Hynes (2024). The integration of human and non-human actors to advance healthcare delivery: unpacking the role of actor-network theory, a systematic literature review. BMC Health Serv. Res. 24:256. doi: 10.1186/s12913-024-11866-4 39497065 PMC11536900

[ref30] Samson P. L. Nicholas D. Gloeckler T. Samek D. Zawaski N. (2025). From theory to praxis: conceptualizing communities of practice for social workers in healthcare settings. Soc. Work Health Care 64, 222–241. doi: 10.1080/00981389.2025.2552661, 40875613

[ref31] Sawik B. Tobis S. Baum E. Suwalska A. Kropińska S. Stachnik K. . (2023). Robots for elderly care: review, multi-criteria optimization model and qualitative case study. Healthcare 11:1286. doi: 10.3390/healthcare11091286, 37174828 PMC10178192

[ref32] Sayes E. (2014). Actor–network theory and methodology: just what does it mean to say that nonhumans have agency. Soc. Stud. Sci. 44, 134–149. doi: 10.1177/0306312713511867, 28078973

[ref33] Statistics Canada (2024) The older people are all right. Available online at: https://www.statcan.gc.ca/o1/en/plus/7059-older-people-are-all-right (Accessed November 1, 2025)

[ref34] Tuisku O. Johansson-Pajala R. M. Hoppe J. A. Pekkarinen S. Hennala L. Thommes K. . (2023). Assistant nurses and orientation to care robot use in three European countries. Behav. Inform. Technol. 42, 758–774. doi: 10.1080/0144929X.2022.2042736, 37339054

[ref35] United States Census Bureau (2020) Demographic turning points for the United States: population projections for 2020 to 2060 Washington, DC. U.S. Department of Commerce. Available online at: https://www.census.gov/content/dam/Census/library/publications/2020/demo/p25-1144.pdf (Accessed May 1, 2026)

[ref36] West C. A. Petrovskaya O. (2025). Social theory in nursing scholarship, from humanism to post-humanism: revisiting S. Nairn on the structure–agency debate. Nurs. Philos. 26, e70040–e70049. doi: 10.1111/nup.70040, 40981532 PMC12452806

[ref37] Wong J. Young E. Hung L. Mann J. Jackson L. (2023). Beyond plan-do-study-act cycle—staff perceptions on facilitators and barriers to the implementation of telepresence robots in long-term care. BMC Health Serv. Res. 23:772. doi: 10.1186/s12913-023-09741-9, 37468953 PMC10357815

[ref38] World Health Organization (2025) Ageing and health. Available online at: https://www.who.int/news-room/fact-sheets/detail/ageing-and-health (Accessed May 1, 2026)

